# Retracted: Yu, L., Li, H., Liu, W., Zhang, L., Tian, Q., Li, H., & Li, M. (2021). miR‐485‐3p serves as a biomarker and therapeutic target of Alzheimer's disease via regulating neuronal cell viability and neuroinflammation by targeting AKT3. *Molecular Genetics & Genomic Medicine*, *9*(1), e1548. https://onlinelibrary.wiley.com/doi/10.1002/mgg3.1548

**DOI:** 10.1002/mgg3.1813

**Published:** 2021-12-06

Retraction: Yu, L., Li, H., Liu, W., Zhang, L., Tian, Q., Li, H., & Li, M. (2021). miR‐485‐3p serves as a biomarker and therapeutic target of Alzheimer's disease via regulating neuronal cell viability and neuroinflammation by targeting AKT3. *Molecular Genetics & Genomic Medicine*, *9*(1), e1548. https://doi.org/10.1002/mgg3.1548. The above article, published online on 21 November 2020 in Wiley Online Library (wileyonlinelibrary.com), has been retracted by agreement between the authors, the journal Editor in Chief Dr. Suzanne Hart, and Wiley Periodicals LLC. The retraction has been agreed because the authors have been unable to reproduce the differences in serum miR‐485‐3p expression in patients with Alzheimer’s disease. Thus, the conclusion that serum miR‐485‐3p is a diagnostic biomarker of Alzheimer’s disease may not be reliable.

